# Substrate and Passivation Techniques for Flexible Amorphous Silicon-Based X-ray Detectors

**DOI:** 10.3390/s16081162

**Published:** 2016-07-26

**Authors:** Michael A. Marrs, Gregory B. Raupp

**Affiliations:** 1 Flexible Electronics and Display Center, Arizona State University, Tempe, AZ 85284, USA; 2 School for Engineering of Matter, Transport, and Energy, Arizona State University, Tempe, AZ 85287, USA; raupp@asu.edu

**Keywords:** flexible electronics, flexible displays, flexible X-ray detectors, amorphous silicon PIN diodes, passivation

## Abstract

Flexible active matrix display technology has been adapted to create new flexible photo-sensing electronic devices, including flexible X-ray detectors. Monolithic integration of amorphous silicon (a-Si) PIN photodiodes on a flexible substrate poses significant challenges associated with the intrinsic film stress of amorphous silicon. This paper examines how altering device structuring and diode passivation layers can greatly improve the electrical performance and the mechanical reliability of the device, thereby eliminating one of the major weaknesses of a-Si PIN diodes in comparison to alternative photodetector technology, such as organic bulk heterojunction photodiodes and amorphous selenium. A dark current of 0.5 pA/mm^2^ and photodiode quantum efficiency of 74% are possible with a pixelated diode structure with a silicon nitride/SU-8 bilayer passivation structure on a 20 µm-thick polyimide substrate.

## 1. Introduction

### 1.1. Flexible Electronics

Flexible electronics are becoming more prevalent as previously developed flexible active matrix display technology is being implemented to produce a wide variety of photo-based biomedical sensing devices [1,2], including flexible X-ray detectors [3]. The primary immediate benefit of switching from conventional display glass to flexible substrates in digital radiography is the cost savings associated with the elimination of the significant ruggedization that must be incorporated to limit detector breakage.

Portable radiography has seen expansive growth into non-medical markets, such as security imaging and non-destructive testing for integrity analysis (i.e., looking for cracks in pipelines or inspection of aircraft structures). Though some portable X-ray panels do exist, they are comprised of glass thin film transistor (TFT) panels, and are rather bulky due to the ruggedization required to protect the costly panel from breakage. Given the cost of these panels, it would be advantageous to have a more rugged system that would be less prone to breakage and be much lighter for mobile users to carry. 

Along with being both portable and lightweight, a digital X-ray panel with some degree of flexibility or bendability is also an appealing medical diagnostic or industrial imaging tool, especially if the overall digital X-ray detector thickness can be also minimized. A paramedic would be able to easily slide a thin and slightly flexible digital X-ray detector panel directly underneath the victim of a car accident at the scene, or an inspector could wrap the X-ray detector around a possibly cracked oil pipeline. 

The relatively rapid development of flexible X-ray detectors and imagers has been enabled by the fact that existing active matrix thin film transistor flexible display technology can be easily ported to amorphous silicon (a-Si)-based indirect digital X-ray detectors, which have a similar underlying platform structure to liquid crystal displays (LCD). The principal new challenge to be addressed is flex-compatible fabrication of photodiodes in series with the thin film transistor at each pixel.

### 1.2. X-ray Source and Detector Structure and Operation

An X-ray source typically consists of a vacuum tube in which high voltage (i.e., 30–150 kV) electrons are accelerated into a metallic anode. The accelerated electrons pass close to the nuclei of the target material, or strike an inner shell electron in some cases, and produce an X-ray as the electron is slowed considerably by the opposing charge of the nucleus [4]. In the event of an inner electron shell collision, an electron from the outer shell moves into the vacancy in the inner shell and gives off an X-ray with energy that is characteristic of the anode material. The energy of the X-rays can be attenuated by a filter (which can be aluminum or carbon), producing a target energy range of 70–80 keV.

X-rays from the source pass through the patient or device under examination, with some of the X-rays attenuated by the nuclei of the intervening material. At the kilovolt energies typically employed by medical X-ray detectors, attenuation is usually the result of photoelectric absorption or Compton scattering. Photoelectric absorption produces meaningful data because an X-ray photon is absorbed and releases an electron from the absorbing atom. The X-ray absorption is proportional to the atomic number, the density, and the thickness of the absorbing media [5]. As an example, bone—which has a higher effective atomic number and is relatively dense—will absorb more X-rays than soft tissue, which results in a contrast difference in the completed radiograph. X-rays that do pass through the patient are absorbed by the detector.

A flat panel digital X-ray detector is usually classified as direct conversion or indirect conversion, depending on the process for converting the incident X-rays into charge. Direct conversion detectors utilize a photoconductive layer of amorphous selenium, which absorb the X-ray and produce an electron/hole pair. When a bias is applied across the selenium, the generated charge carriers are pulled towards the electrodes. When an X-ray is absorbed and generates an electron/hole pair, the charge carrier is drawn to the opposing electrode with limited scattering in comparison to indirect detection techniques [6]. However, the amorphous selenium has a smaller capture cross-section in comparison to the cesium iodide (CsI) screens used in indirect conversion, thus requiring the patient to be exposed to a higher dosage of X-rays to achieve the same resolution. In addition, the selenium film is usually 0.25–1.0 mm thick, requiring a large voltage on the order of 10 kV across the selenium to provide a sufficient electric field to extract the incident X-ray-generated charge carriers [6].

The basic components of an indirect digital X-ray detector include a scintillator phosphor conversion film that converts the incident X-rays into visible photons, a photodiode—usually amorphous silicon with organic photodiodes as an emerging competing technology—that detects the light emitted from the X-ray phosphor conversion film and converts the light into an electric charge, and a thin film transistor (TFT) that acts as a switch for the readout of the charge stored in the photodiode in between readout. In that sense, the operation of a digital X-ray detector can be compared to a large digital camera. The scintillator is typically composed of high capture cross section materials like cesium or the rare earth elements doped with a phosphor whose peak emission is in the visible spectrum usually between 500 and 550 nm for detectors manufactured with a-Si photodiodes. The a-Si photodiode is operated under reverse bias between −2 and −5 V so that the photocurrent dominates the total current flowing through the photodiode. The a-Si is grown to a thickness of 1.0 to 1.2 µm, which is more than adequate to capture most of the incident green light produced by the scintillator. The TFTs are laid out in a grid similar to an LCD display, where the gate lines are scanned sequentially and the charge stored on the source line is read out at the edge of the array.

### 1.3. Flexible Indirect X-ray Detectors

The simplest photodiode fabrication approach is to pattern the n-Si layer of the photodiode at each pixel, and to blanket deposit the i-Si, p-Si, and transparent conductor top metal contact continuously over the entire array. A major advantage of this full fill factor approach is that the entire pixel is covered by the i-Si absorption layer, thus eliminating any “dead” spots in the pixel where photon strikes will not be detected. This full fill factor approach also produces the smallest pixel size (and hence highest resolution) for a given set of design rules. For example, for X-ray detectors currently fabricated at the Flexible Electronics and Display Center, we produce 50 ppi full fill factor arrays, compared to much larger 83 ppi for pixelated diode arrays.

**Figure 1:**
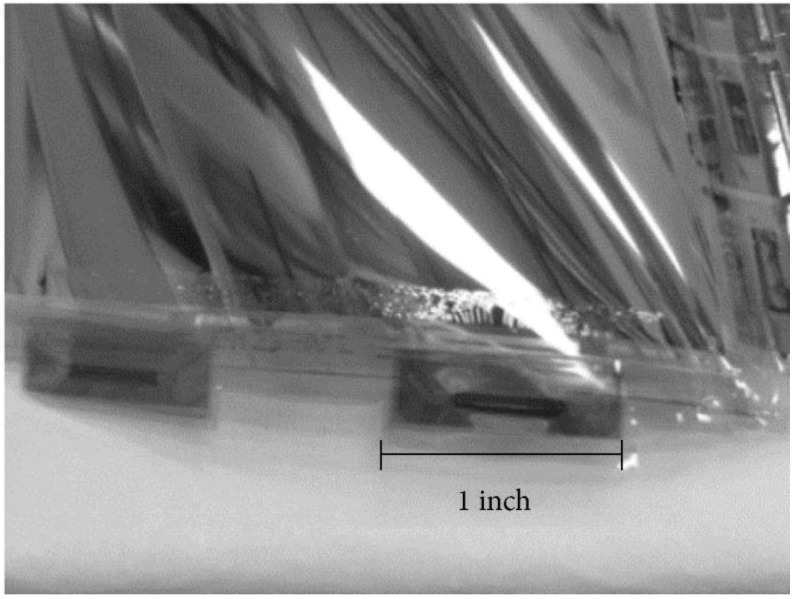
Full fill factor digital X-ray detector backplane showing stress related failure due to high intrinsic film stress in the i-Si layer.

There are some significant processing drawbacks to the full fill factor design that are exacerbated by the use of polyethylene naphthalate (PEN) as a flexible substrate. Since there is a large coefficient of thermal expansion (CTE), mismatch between PEN and amorphous silicon (13 ppm/°C versus 3 ppm/°C), coupled with the high intrinsic compressive film stress of the i-Si layer detectors, arrays fabricated with the full fill factor approach have a tendency to curl significantly after post-fabrication debonding of the flexible substrate. In addition, the deposition process for the amorphous silicon diode layers takes place at 200 °C, which is near the melting temperature of the PEN (240 °C), and can take upwards of one hour to deposit the requisite 1.2 µm-thick film. The extended exposure at the elevated temperature of the PECVD (plasma enhanced chemical vapor deposition) process risks embrittling the PEN and ruining the detector. In spite of these drawbacks, full fill factor X-ray detectors on PEN substrates have been successfully demonstrated by multiple groups [3,7,8]. Previous results from the Flexible Electronics and Display Center are shared below in Figure 1. If the i-Si deposition process is not tightly controlled, stress-related failure, as shown in Figure 1, may occur.

In spite of these drawbacks, amorphous silicon photodiodes are more desirable for large area detectors than direct conversion amorphous selenium detectors because the amorphous silicon is significantly thinner (1.2 µm [3] vs. 100 µm [7]), enabling a tighter bending radius. Moreover, PECVD amorphous silicon can be deposited with better uniformity over large areas than the thermally evaporated amorphous selenium. Amorphous silicon photodiodes are preferable over organic photodiodes due to the air and moisture instability of organic photodiodes under irradiation, which requires a sufficiently robust moisture and oxidation barrier to prolong the life of the detector [9].

Another method for alleviating stress in the entire film stack is to use a flexible substrate whose coefficient of thermal expansion is a closer match to that of the deposited thin films. Polyimides are a class of flexible substrate materials receiving considerable attention because of their compatible thermal properties. The main advantages of polyimides over PEN as a substrate are that the coefficient of thermal expansion for polyimide is roughly four times smaller than PEN, while the safe operating temperature is roughly 200 °C higher. A comparison of the thermal properties of HD Microsystems PI 2611 [10], a polyimide, DuPont Teonex Q51 [11], and silicon [12,13] are shown below in Table 1.

The increased operating temperature of the polyimide would allow for safe processing of a-Si at temperatures above 275 °C, which is a typical deposition temperature for high quality TFT and PIN diode a-Si films, without the risk of significant thermal damage to the substrate. In addition, the coefficient of thermal expansion of the PI 2611 is within 0.4 ppm/°C of silicon, meaning that it is less likely that intrinsic stress associated with a-Si deposition will cause polyimide substrates to physically curl once they are debonded from the carrier.

## 2. Materials and Methods

### 2.1. Substrate Preparation: Polyethylene Naphthalate (PEN) Bond/Debond

**Figure 2:**
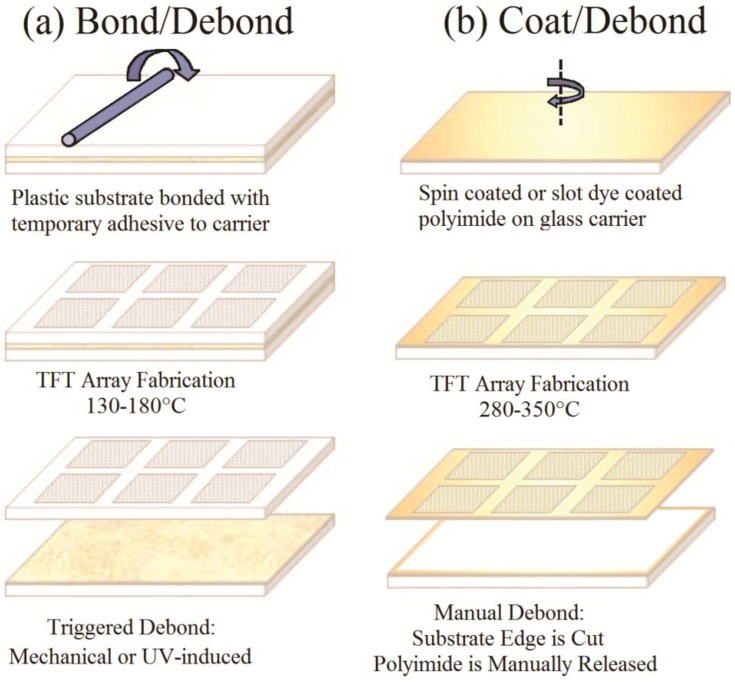
Bond/debond and polyimide coat/debond process flow. TFT: thin film transistor.

The bond/debond (or temporary bonding process) allows the processing of a flexible substrate bonded temporarily to a rigid carrier in conventional semiconductor or flat panel display (FPD) process tools that are built to handle rigid Si or glass substrates. The basic process flow for the bond process is shown in Figure 2a [14], where it is compared to the polyimide cast and debond process, which is described in the next sub-section. A temporary adhesive is spin-coated on to a rigid carrier. If the adhesive is thermally cured, a bake usually follows the spin process. The flexible substrate, in this case polyethylene naphthlate (PEN), is then mounted to the adhesive-coated carrier using a roll laminator. The adhesive is cured either by UV exposure, by baking, or with a combination of both.

The rigid carrier suppresses the bowing of the flexible substrate during processing to provide the requisite dimensional stability during device fabrication. Following device fabrication, the flexible substrate can be debonded from the rigid carrier to yield a flexible electronics device (display, sensor array, detector).

The main advantage of the bond/debond process is that the process requires little additional investment to an already existing display or semiconductor fabrication facility and is scalable to larger area substrates without significant alterations to the process. The main disadvantage of the bond/debond process is related to controlling substrate deformation (i.e., warp and bow), which can cause wafer handling and pattern alignment issues, substrate distortion, and in extreme cases delamination of the flexible substrate during processing. However, Haq, et al. have demonstrated a suitable distortion-free bond/debond process with controlled warp/bow that is capable of effective processing up to 200 °C [15].

### 2.2. Substrate Preparation: Polyimide Cast and Debond Process

The polyimide cast and debond process is an attractive alternative to the EPLaR (Electronics on Plastic by Laser Release) [16] process, where slot die coating is used to cast polyimide as the base substrate onto a display glass substrate, as shown in Figure 2b. The adhesion strength of the polyimide is selectively modified at the edge of the carrier by applying an adhesion promoter such as VM652 from HD Microsystems. The increased adhesion strength at the edge significantly reduces edge-initiated debonding. However, the polyimide-to-glass adhesion strength has been tailored to be significantly smaller than adhesion strength of the polyimide to the adhesion promoter, thus eliminating the need for excimer laser melting of an interfacial layer to release polyimide, as in the EPLaR process. After the polyimide is cured at 375 °C in an inert atmosphere, a PECVD silicon nitride (SiN) barrier film is applied to protect the polyimide from future processing steps and to reduce the water vapor transmission rate (WVTR) of the polyimide. The TFT and PIN diode processing steps can proceed once the barrier is deposited.

After the device layers are fabricated, the backplane is cut inside the adhesion promotion layer picture frame and is removed manually. The adhesion promotion layer remains bonded to the glass carrier substrate along with a polyimide picture frame, which can now be reclaimed if desired. The curl of the polyimide substrate after debond depends on the formulation of the polyimide spin/spray casting solution and adhesion layer. The control of the post-debond curl is the biggest challenge of the polyimide process.

### 2.3. PIN Diode Processing and Device Structure

**Figure 3:**
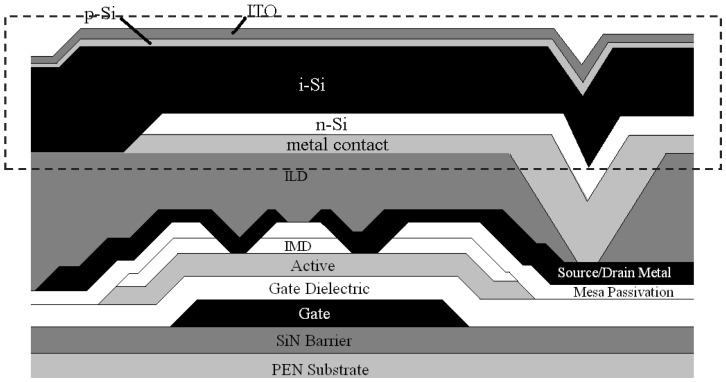
Full fill factor PIN photodiode connected in series to a TFT. The interlayer dielectric (ILD) is a combination of SU-8 (MicroChem) and SiN. The active layer is amorphous Si and the gate dielectric, mesa passivation, and intermetal dielectric (IMD) are SiN.

Figure 3 is a cross-sectional schematic that illustrates the basic full fill factor pixel design. The grey dashed line box highlights the PIN diode layers. The current full fill factor design places the PIN diode over the top of the TFT and uses an indium tin oxide (Siommon electrode for the V_bias_ connection on the p-doped side of the PIN diode. The i-Si, p-Si, and ITO blanket the entire X-ray detector array, and are etched outside the array in the field and over the driver connections. Processing of the full fill factor design was described previously [3,17]. The maximum processing temperature for the device is 200 °C.

Although the full fill factor design provides a smaller-area, higher resolution pixel when subjected to the same design rules as alternative pixelated structures [18], there are some potential disadvantages as well. For example, since the i-Si is blanket deposited across the array, it imparts more stress on the substrate system than if the i-Si layer were patterned. In addition, there is also potential for crosstalk between the elements in the arrays, since photons absorbed physically over a certain pixel could instead be read out by a neighboring pixel because the i-Si film is continuous over the entire array.

**Figure 4:**
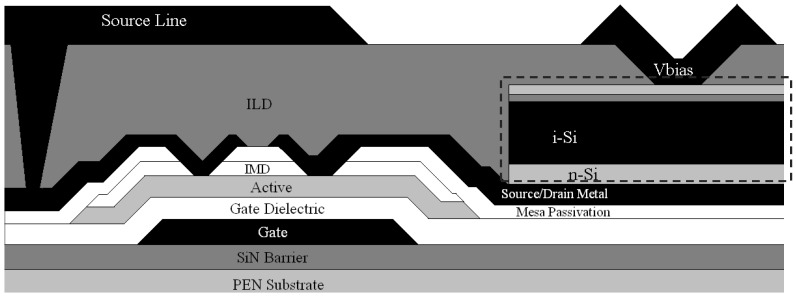
Patterned PIN photodiode connected in series to a TFT.

Figure 4 is a cross-sectional schematic that illustrates the basic design for the alternative pixelated diode approach. The grey dashed line box again highlights the PIN diode layers. Pixelating the photodiode requires the V_bias_ connection to the p-doped side of the diode to be made using a subsequently deposited metal deposition. The readout lines can also be moved to this subsequent deposition step and then connected down to the TFT through a via at each pixel. This modification enables the maximum separation between the gate lines and the readout lines, which reduces the parasitic capacitance (noise) between the two. The separation of the diode and the TFT reduces the area available for detection by approximately 15% in a detector with a 207 µm by 207 µm pixel compared to the full fill factor design present in Figure 3 with the same pixel area.

The patterned PIN diode structure replaces the ITO V_bias_ connection with any opaque metal of choice. If a sputtered aluminum/1 wt% silicon alloy is used, the resistivity difference between the sputtered aluminum/silicon alloy and ITO is over a factor of 100 (4.1 µΩ-cm vs. 528 µΩ-cm), which should provide an immediate benefit by reducing the series resistance in the device. The ITO thickness and thus the sheet resistance is governed by optical constraints as well as electrical constraints. Specifically, the ITO layer needs to act as an anti-reflection coating at the target wavelength for the detection of incident light. In the case of a digital X-ray detector using a CsI scintillator, the target light wavelength for detection is the peak emission wavelength of the scintillator (roughly 525 nm). The ideal ITO thickness to produce minimum reflection at this wavelength is approximately 65 nm thick, as calculated from Snell’s Law. However, the V_bias_ connection in the pixelated PIN diode structure depicted in Figure 4 does not have such optical constraints because the layer is so thick that it is sufficiently opaque to most visible light and only covers a small portion of the photodiode. This feature allows the separate control of the electrical and optical properties of the V_bias_ connection.

By using the aluminum/silicon alloy as the V_bias_ connection over ITO, the series resistance can be significantly reduced while still maintaining the optical properties necessary to maximize photon absorption in the photodiode. Series resistance is a parasitic, power consuming parameter whose effect on the diode performance is illustrated by equation (1), which is a modification of the ideal diode model:

(1) $$\;I_{D}=I_{S}\left(e^{\frac{q\left(V_{bias}-I_{D}R_{s}\right)}{nkT}}-1\right)$$

where *I_D_* is the diode current, *I_S_* is the reverse saturation current, *q* is the charge of an electron, *V_bias_* is the voltage applied across the diode, *R_s_* is the series resistance, *n* is the diode ideality factor, *k* is Boltzmann’s constant, and *T* is the temperature. The main contribution to series resistance comes from the diode contact resistance, bulk resistance of the diode layers, and the sheet resistance of the top metal layer [19]. The deleterious effects of series resistances are most noticeable at voltages near the open circuit voltage of the photodiode [20] as a deviation in the slope of the I–V curve from the vertical affecting the quantum efficiency in extreme cases [19].

### 2.4. PIN Diode Test Setup

#### 2.4.1. L-I-V Sweeps of 1 mm^2^ Diodes

PIN diode test structures with an area of 1 mm^2^ were separately fabricated side-by-side with the main arrays to test and monitor the performance of the full fill factor and pixelated PIN diodes. A probe station fitted with a Keithley 4200 Semiconductor Characterization System was used to characterize the diode performance. The probe card on the probe station is fitted with a green LED illuminator (peak emission wavelength of 520 nm), which generates an irradiance 102 W/m^2^ at the device surface. The diode is swept from −5 V to +1 V in the dark and again with the illuminator set to maximum current. The fill factor, open circuit voltage (V_OC_), and short circuit current (I_SC_) are extracted from the irradiated curve. The dark characteristics are fitted to the ideal diode equation, which yields the diode saturation current and the ideality factor. In addition, the minimum dark current, the dark current at −5 V, and the dark current at +1 V are extracted to indirectly track the shunt resistance and series resistance, as well as the magnitude of the dark current. After the sweeps are completed, the diode is held at −5 V in the dark as the prober continues to read the current an additional 120 times, with each reading commencing once the previous reading has collected sufficient charge to be safely above the noise floor threshold of the tester.

#### 2.4.2. Diode Array Testing

A custom-built array test system with readout chips was built in-house to test the arrays. The tester was customizable so that arrays as small as 128 by 240 pixels or as large as 1024 by 720 pixels could be tested. The gate on time was set to 20 µs to allow for sufficient discharge of the photodiodes and the charge collected was recorded to an image with 12 bit range greyscale.

## 3. Results

### 3.1. L-I-V Sweep Comparison between Blanket and Pixelated Photodiodes

**Figure 5:**
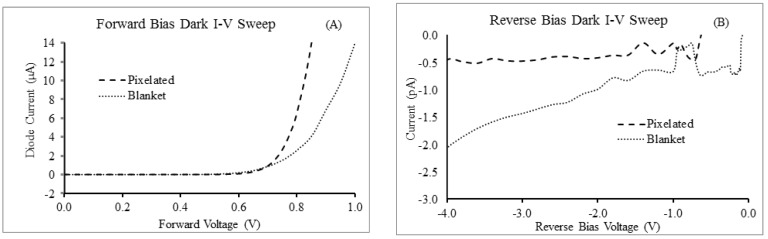
(**A**) Forward and (**B**) Reverse dark I-V sweep comparison between blanket and pixelated PIN diodes.

Figure 5A compares dark I-V characteristics of 1 mm^2^ diodes fabricated with a blanket PIN diode structure with ITO top contact and no passivation (structure shown in Figure 3), with the characteristic behavior of diodes that are passivated and utilizing a metal strap as the top V_bias_ contact (structure shown in Figure 4). The passivated, pixelated PIN diode performs better than the blanket structure under both forward bias and reverse bias. The superior performance under forward bias is likely attributable to the reduced series resistance, which is expected to dominate the I-V characteristic as the voltage increases above 0.5 V. The primary advantage to decreasing the series resistance is to reduce the image lag and prevent inverse shadowing. A lower resistance allows charge to be drained faster and prevents the applied bias voltage from being pulled down when reading highly charged pixels.

Under reverse bias operation—under which a photodiode will spend most of its operating lifetime—the leakage current for the pixelated diode is lower than the blanket coated diode and stays relatively flat with increasingly negative applied voltage (Figure 5B).

When irradiated with green light at an irradiance of 102 W/m^2^, the quantum efficiency is calculated to be 78% for the blanket device and 74% for the pixelated device. The reduced quantum efficiency of the pixelated device is due to reflection at the passivation interfaces.

### 3.2. Effect of the Photodiode Passivation Layer

#### 3.2.1. Dark Current vs. Time Stability

**Figure 6:**
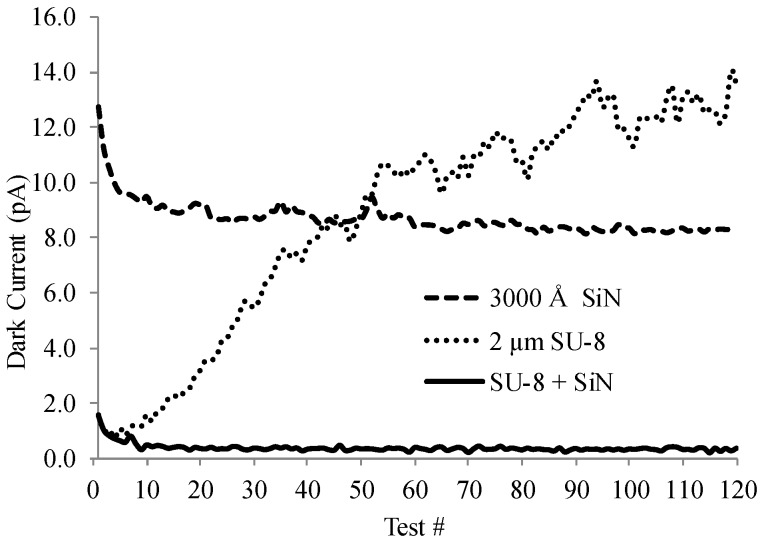
Dark current vs. test number for three different photodiode passivation schemes. The time interval between tests was not consistent due to the low current of the diodes and the charge integration time required to measure the current.

The nature of the passivation layer(s) affects the final dark current characteristics. When no passivation is used, the diodes are so leaky that it is difficult to consider them “diodes”. In the example presented in Figure 5, the passivation is a two-layer stack comprising 2.0 µm of spin on SU-8 polymer and 300 nm of PECVD-deposited SiN. If only the SU-8 polymer is applied as the passivation layer, the initial dark current behavior is greatly improved. However, the dark current begins to degrade during continuous operation under reverse bias, as shown in Figure 6.

Figure 6 further shows that if only SiN is used as the passivation material, the initial dark current is significantly larger (~10 times) than the initial dark current for SU-8-coated diodes. However, the dark current exhibits a monotonically-decreasing current with time (eventually leveling off to a near constant value), consistent with thermally generated leakage associated with the depopulation of defects/traps in the i-Si [21]. This time-dependent behavior suggests that the PECVD SiN film deposition process may damage the exposed PIN diode layers, potentially increasing the number of surface trap sites. If only SU-8 is used as the passivation, the dark current increases with time, suggesting that SU-8 by itself may be an inadequate atmospheric contaminant barrier, and that atmospheric contaminants diffuse to the i-Si surface and react. The best results are observed when SU-8 is coated first to avoid photodiode damage, followed by SiN deposition.

#### 3.2.2. Effect of Diode Shape

The effect of surface generation/recombination can be explored by looking at the performance of photodiodes with the same detection area (i.e., the surface area of the side of the diode facing the incident light), but with a different perimeter. Square diodes have the minimum perimeter possible for a given area and thus have the smallest surface area of i-Si sidewalls exposed. Diodes with a rectangular shape have a larger area for potential surface recombination and generation to occur and thus should have a higher dark current than a square diode. To illustrate this point, two different diode geometries were investigated. Square Diode A has a 1 mm by 1 mm square cross-section exposed to the incident light, while rectangular Diode B has a 2 mm by 0.5 mm rectangular cross-section exposed to the incident light. Both diodes have the same capture area (1 mm^2^).

A *t*-test reveals a small but statistically significant difference in the logarithm of the dark current, with the rectangular diodes having greater than two times the dark current of the square diodes.

#### 3.2.3. Effect of a Backside Guard Ring on Humidity Sensitivity

**Figure 7:**
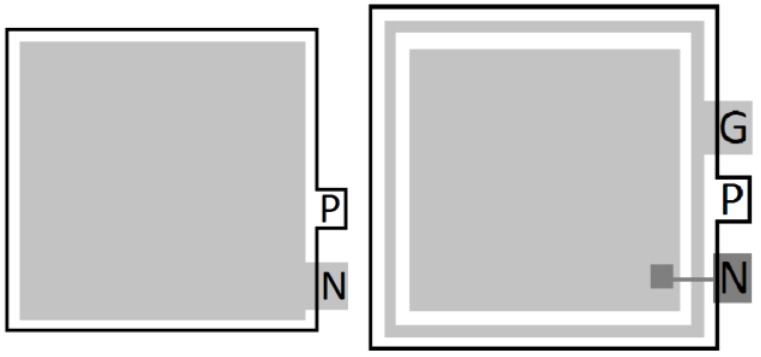
Top-down drawing comparing an unguarded PIN diode with only N and P terminals (**Left**); and guarded PIN diode structure with added guard ring (G) in addition to the N and P terminals (**Right**).

The effect of the diode edge can also be illustrated by testing a structure where there is a diode completely surrounded by another diode as a protective backside guard ring, as shown schematically in Figure 7. The backside guard ring reduces the electric field at the edge of the device and prevents the depletion region from reaching the edge of the diode, thus mitigating surface leakage and enabling the capture of carriers generated outside of the active area [22]. The beneficial effects of the guard ring can be enhanced by grounding the n side of the diode, as opposed to having it float.

**Figure 8:**
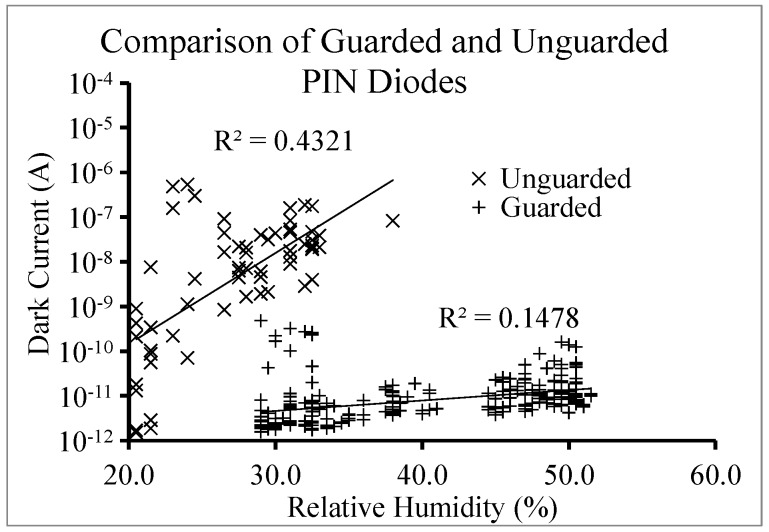
Comparison of the dark current vs. relative humidity for guarded and unguarded PIN diodes.

Figure 8 compares dark sweeps from diodes with and without guard rings, and clearly shows that the devices with the guard ring demonstrate a substantial reduction in the dark current. In addition, devices with the guard ring are less sensitive to environmental conditions, specifically humidity.

#### 3.2.4. Array Performance

**Figure 9:**
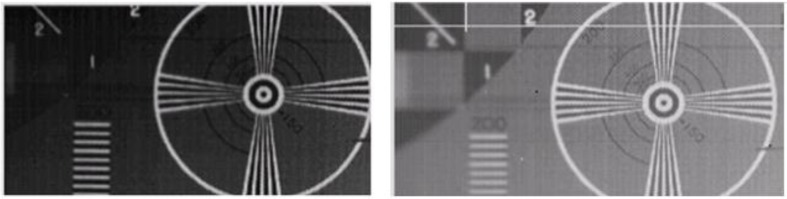
Full fill factor blanket i-Si photodiode array (**Left**) compared to pixel pattern photodiode array (**Right**).

An advantage of the pixelated PIN diode approach is that the i-Si absorbing layer is not continuous over the entire array, making it extremely difficult for photons absorbed over one pixel to be read out by a neighboring pixel. This reduction in cross-talk between neighboring pixels results in increased contrast and improved resolution. Figure 9 shows a comparison between an array with a blanket i-Si layer and a pixel patterned i-Si when exposed to the same irradiance and with the same gate on time for the TFT array. The pixel patterned array demonstrates a notably higher contrast, thereby suggesting that it operates with a lower detection threshold.

#### 3.2.5. PIN Diode Film Cracking

**Figure 10:**
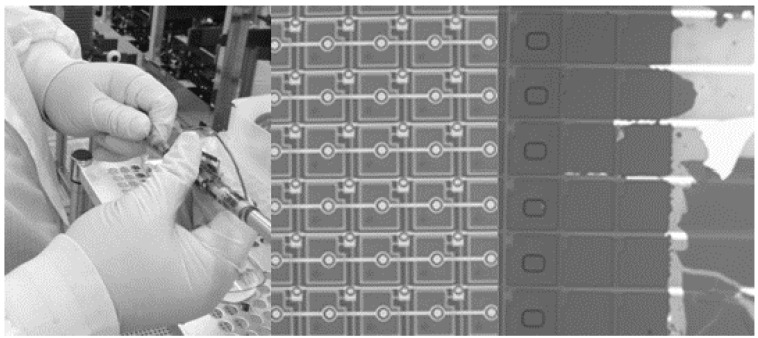
Flexible photodiode array wrapped around a 3/8” stainless steel pipe (**left**), microscope image of pixelated PIN diode structure (**center**), and full fill factor PIN diode structure (**right**) after wrapping around 3/8” stainless pipe.

The pixelated PIN diode structure relieves the overall film stress of the entire device. With the blanket photodiode structure, the PIN diode layers have a tendency to crack and peel when the detector array is curled. Arrays with both the full fill factor blanket photodiode structure and the pixelated PIN diode structure were curled around a 3/8” stainless steel pipe, as shown in Figure 10.

Of the 24 arrays mechanically tested with the pixelated photodiode structure, none demonstrated any physical damage other than the embedding of particles from the pipe and manual handling of the arrays. Of the 24 arrays tested with the full fill factor PIN diode structure, 17 demonstrated significant cracking and flaking of the PIN diode layers, as shown in Figure 10. 

#### 3.2.6. Effect of the Substrate: PEN vs. Polyimide

**Figure 11:**
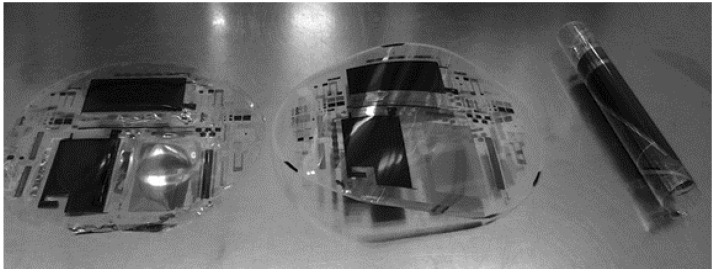
Curl comparison of low coefficient of thermal expansion (CTE) polyimide (**Left**), PEN (**Middle**), and high CTE polyimide (**Right**) debonded.

Significant stress can be imparted on the polyimide if the peel strength is above 80 g/inch, resulting in the elongation or stretching of the polyimide. The polyimide monomer formulation is a factor in the coefficient of thermal expansion for the final film. If the peel strength is too high or the coefficient of thermal expansion is significantly different from the thin films deposited, significant curl will be present in the debonded polyimide, rendering the flexible X-ray detector unusable. Using HD microsystems 2611 series polyimide with an appropriate adhesion promotor at the edge of the rigid carrier substrate can yield a flexible substrate that is easily debonded post process and does not exhibit significant curl. The curl of the 2611 series polyimide is lower than PEN. A comparison of a debonded PEN X-ray detector, a debonded polyimide X-ray detector with large peel strength, and a debonded polyimide X-ray detector with appropriate peel strength and minimal coefficient of thermal expansion mismatch is shown in Figure 11.

Once the polyimide substrate is debonded, the user can be quite rough with the substrate and not be concerned with film cracking or peeling. One must be very cautious and slow while debonding the PEN, as perturbations during the debond process can cause the inorganic PIN diode layers to buckle under the sudden change in applied peeling force as the residual stress in the PEN is released.

The maximum processing temperature of the TFT and PIN diode processing is 200 °C, which is 20 °C above the recommended continuous operation temperature, but 69 °C below the melting temperature listed in Table 1. However, at 210 °C and above, the PEN crystallizes, leading to significant shrinkage, and is prone to fracture during the debond process [23]. In this dynamic situation, even minor perturbations in the temperature of the TFT and PIN diode thin film deposition processes could significantly affect the structure of the bonded PEN, potentially leading to film buckling during the debond process.

In addition to the reduced curl and the improved film adhesion post debond, another advantage of using polyimide over PEN is the significantly higher glass transition temperature of the polyimide (360 °C vs. 121 °C, respectively). The higher glass transition temperature allows for a higher deposition temperature during the PECVD steps. The added degree of freedom allows for a higher quality finished product, as the higher deposition temperature can lead to a lower dark current, higher quantum efficiency, and higher fill factor for the PIN diode films.

Simply increasing the deposition temperature of the PIN diode films from 200 °C to 250 °C, without making any other changes to the other deposition parameters, results in a significant increase in the quantum efficiency of the photodiodes at 520 nm from 57.8% to 61.8% when using a green LED light source with measured irradiance of 100 W/m^2^. The increased deposition temperature of the PIN diode layers results in a denser, higher quality film that has fewer trap sites available to grab photogenerated electrons and holes before leaving the diode.

## 4. Conclusions

The benefits of a pixelated PIN diode structure were evaluated in comparison to full fill factor diodes with respect to flexible X-ray detectors. In addition to demonstrating lower dark current that is more stable with time and having less cross-talk between pixels, the pixelated structure is more robust with respect to flexing and bending of the substrate. The pixelated structure does not require additional masks in comparison to the blanket diode structure, but enables the passivation of the diode, allowing for the mitigation of surface edge-related leakage and reduction of the overall dark current of the device. The biggest downside to the structure is the extra layout spacing necessary to place the PIN diode next to the TFT, as opposed to on top of the TFT. However, this layout concern is only an issue with flexible detectors that must have a resolution less than 83 dpi.

Further benefits can be realized by utilizing polyimide as a substrate. HD Microsystems 2600 series polyimide has the right combination of low coefficient of thermal expansion and moderate adhesion strength to glass to enable a successful, low stress debond with minimal curl in the polyimide. In addition, the higher melting temperature of the polyimide allows for higher temperature PIN diode processing, leading to improved diode performance.

## Figures and Tables

**Table 1:** Comparison of structural, thermal, and electrical properties of polyimide, polyethylene naphthalate (PEN), and silicon.

	HD Microsystems PI 2611	DuPont Teonex Q51 PEN	Silicon	Units
Tensile Strength	350	274.6	7000	MPa
Glass Transition Temperature	360	121	-	°C
Continuous Operation Temperature	-	180	-	°C
Melting Temperature	620 (decomposes)	269	1415	°C
Coefficient of Thermal Expansion	3	13	2.6	ppm/°C
Dielectric Constant	2.9	3	11.9	
